# Minimally Invasive Surgical Techniques in the Treatment of Appendicitis: A Narrative Review

**DOI:** 10.7759/cureus.99125

**Published:** 2025-12-13

**Authors:** Mohummed S Alrayes, Mohammed Ahmad Altawili, Ahmed Nasser A Almutawah, Joud Abdulhamid S Alhassun, Abdulelah Mabruk B Alharthi, Omar B Alsheikh Alshahrani, Naif Ayidh Q Alharthi, Omar Mohammed Almithn, Abdulrahman Sultan Abdullah Alahmari, Saleh Waleed Bubshait, Moath Mubarak A Alharthi

**Affiliations:** 1 General Surgery, King Faisal Specialist Hospital and Research Centre, Tabuk, SAU; 2 General Surgery, King Faisal University, Hofuf, SAU; 3 General Practice, Albadai General Hospital, Al Badʿ, SAU; 4 General Practice, King Faisal University, Hofuf, SAU; 5 General Surgery, College of Medicine, Bisha University, Bisha, SAU; 6 General Surgery, Faculty of Medicine, Zagazig University, Zagazig, EGY

**Keywords:** appedicitis, endoscopic retrograde appendicitis therapy, laparoscopic appendectomy, minimally invasive, natural orifice transluminal endoscopic surgery, robotic-assisted appendectomy, single-incision laparoscopy, surgery

## Abstract

Acute appendicitis is the most common abdominal surgical emergency worldwide, conventionally managed through open appendectomy until the advent of minimally invasive techniques. This narrative review gathers current evidence on minimally invasive surgical approaches for appendicitis, such as laparoscopic, single-incision, robotic, natural orifice transluminal endoscopic surgery, and endoscopic retrograde appendicitis therapy. The primary objective was to assess the efficacy, accessibility, and challenges of these techniques while addressing barriers to global implementation and strategies for optimization. Laparoscopic appendectomy is considered the gold standard, offering shorter hospital stays, reduced postoperative pain, and fewer surgical site infections compared to open surgery. Innovations such as single-incision laparoscopy and endoscopic retrograde appendicitis therapy show promise in enhancing cosmetic outcomes and organ preservation but encounter limitations because of technical complexity, costs, and infrastructural requirements, particularly in low-resource settings. Robotic-assisted appendectomy shows better ergonomics and recovery benefits but remains cost-prohibitive for global use. In special populations, like pediatric patients and pregnant women, tailored approaches enhance safety and outcomes. However, significant disparities remain in global access to minimally invasive techniques, caused by economic constraints and training gaps in low- and middle-income countries. In clinical practice, laparoscopic appendectomy is advised where expertise exists, with technique selection guided by patient-specific factors like age, pregnancy, and appendicitis severity. Future efforts must prioritize standardized training, funded equipment, diagnostic algorithms to reduce unnecessary surgeries, and rigorous validation of emerging therapies. Collaborative policy and research initiatives are essential to bridge global differences and achieve evidence-based, minimally invasive management of appendicitis in all populations.

## Introduction and background

Acute appendicitis (AA) is traditionally managed through surgical removal of the inflamed appendix. Historically, treatment evolved from the open appendectomy introduced by Charles McBurney in 1889, a technique that remained the gold standard for nearly a century [1-3]. A major shift occurred in 1983 when Kurt Semm introduced the laparoscopic appendectomy, establishing the foundation for minimally invasive approaches [3]. The laparoscopic technique, performed through three small incisions, was associated with shorter hospital stays, reduced postoperative pain, lower surgical site infection (SSI) rates, and fewer adhesions compared with open appendectomy. These advantages helped pave the way for the broader concept of minimally invasive appendectomy [4]. 

**Figure 1:**
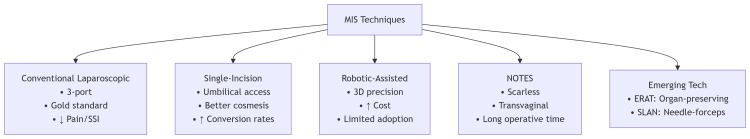
Minimally invasive surgical techniques for appendicitis. The figure highlights the evolution and variety of MIS techniques, from conventional methods to advanced and emerging technologies, noting trade-offs in cost, recovery, visibility, and complexity. MIS: minimally invasive surgery; SSI: surgical site infection; NOTES: natural orifice transluminal endoscopic surgery; ERAT: endoscopic retrograde appendicitis therapy; SLAN: single-port laparoscopic appendectomy using needle-type grasping forceps

Building on this evolution, several minimally invasive techniques have since been developed, including conventional laparoscopic appendectomy (CLA), single-incision laparoscopic appendectomy (SILA), robotic-assisted appendectomy (RAA), mini-laparoscopic appendectomy (LA), natural orifice transluminal endoscopic surgery (NOTES), and endoscopic retrograde appendicitis therapy (ERAT) (Figure 1). These approaches have gained widespread adoption, with reported utilization rates reaching up to 93% in high-resource settings [5]. However, adoption remains inconsistent in developing regions, where variability in infrastructure and resources continues to limit implementation [6]. Importantly, laparoscopic appendectomy can be safely performed by surgical trainees without compromising clinical outcomes or operative times [7].

Despite more than 40 years of refinement, minimally invasive techniques are not universally applicable. Studies report higher rates of intra-abdominal abscess formation in certain patient groups and note technical limitations, particularly in cases of complicated appendicitis [8]. Other documented barriers, including steep learning curves, inconsistent access to equipment, and the financial burden of minimally invasive platforms, also hinder broader uptake worldwide [9].

In this review, we examine the current landscape of minimally invasive appendectomy, highlight the barriers limiting global adoption, and discuss strategies to improve the safe and equitable implementation of these techniques.

Methods

In June 2025, a comprehensive literature search was performed across three electronic databases: PubMed, Scopus, and Web of Science. The search strategy included the following keywords and Boolean combinations: ((Minimally Invasive Surgery) OR (Laparoscopic Appendectomy) OR (Single-Incision Laparoscopic Surgery) OR (Robotic Appendectomy)) AND (Appendicitis). Boolean operators were used to enhance the specificity and sensitivity of the search results. Additional relevant studies were identified by manually reviewing the reference lists of key articles. No filters were applied during the search, except for limiting the results to English-language studies involving human subjects. This study is a narrative review and not a systematic review; therefore, formal inclusion/exclusion criteria, a structured study selection process, and risk-of-bias assessment were not applied.

## Review

Minimally invasive surgical techniques 

Conventional Laparoscopic Appendectomy

LA has become the gold standard in the treatment of AA, quickly overtaking open surgery. As laparoscopic equipment and surgical techniques have improved, CLA has also been used to treat complicated appendicitis, such as gangrenous or perforated appendicitis, as well as peri-appendiceal abscesses [10]. The standard three-port LA remains the most widely used minimally invasive approach. Surgeons typically place a 5-12 mm umbilical port for the laparoscope and two 5 mm working ports in various configurations, maintaining proper triangulation with the appendix at the apex [11,12]. Smaller trocars may shorten hospital stays and postoperative pain since they only make small incisions in the abdominal wall [13], allowing patients to resume their regular activities more quickly. However, this method is more difficult for surgeons who run the risk of a longer surgical procedure and a greater conversion rate [14]. These drawbacks will most likely go away following a suitable learning curve and an improvement in surgical competence. The procedure involves appendix amputation and extraction, with current guidelines discouraging routine peritoneal irrigation or drainage [15]. A two-port variation exists using transabdominal suspension sutures [16]. Laparoscopic operations are less invasive, which leads to faster recovery and less postoperative pain. The technological developments of laparoscopy, which enable a more efficient process, less tissue manipulation, and smaller incisions, could be responsible for the laparoscopic group's shorter operating times [17,18].

Single-incision Laparoscopic Appendectomy

In an effort to find less invasive techniques that yield better cosmetic outcomes, surgical innovation has progressed. The child case in 1992 was the first effort in performing SILA [19]. Esposito described a one-trocar extracorporeal appendectomy through the umbilicus in infants as part of SILA in 1998, although acceptance of this surgery was limited due to early technical limitations [20]. Later, the development of specialized tools, including multi-channel ports, effective laparoscopes, and articulating devices, addressed the aforementioned problems [21]. In 2003, the first SILA was performed on an adult patient [22]. Since then, SILA has been demonstrated to have comparable safety and efficacy compared to CLA [23]. SILA has grown to be one of the most common single-incision laparoscopic procedures because of its safety and feasibility in terms of complications, post-operative pain and recovery, and improved cosmetic outcomes [24-26]. SILA has a learning curve of 5-10 cases, and non-articulating equipment and standard trocars can be used to execute SILA with less expense and specific materials [27]. Even if the operating time is greater, preliminary data indicate that it may be a safe and affordable option, though it has technical obstacles and ergonomic constraints [28].

Robotic-Assisted Appendectomy

Since its military beginnings, the field of surgical robotics has undergone substantial development and is currently used in a variety of medical specialties [29]. Comparative research shows that robotic-assisted procedures perform as well as or better than traditional laparoscopic methods [30,31]. Through improved ergonomics, tremor reduction, high magnification three-dimensional (3D) imaging, and multi-arm control from a console, this technology radically changes the surgeon's experience and may help reduce the learning curve for minimally invasive procedures [32,33].

Building on 35 years of robotic surgical development, RAA is a recent development in minimally invasive surgery [34]. Because of the benefits, including better articulation and wrist-like instrument movement, its use has spread quickly across a number of disciplines [35]. But there is still disagreement over its place in general surgery, especially when it comes to cost-effectiveness [36]. According to National Surgical Quality Improvement Program (NSQIP) data from 2016 to 2019 [37], the use of robotic-assisted approaches has continued to rise steadily across general surgical procedures. The adoption of RA is still limited to less than 0.1% of minimally invasive appendectomies, despite the fact that robotic operations are growing more common (in one database, they increased from 1.8% to 15.1% of general surgeries from 2012 to 2018) [38].

Reported mortality was 2.0% for robotic-assisted surgery versus <0.1% for laparoscopic surgery, which may reflect differences in case selection rather than an intrinsic risk of the approach. Limited added clinical benefit and higher costs compared to laparoscopy remain potential obstacles to broader adoption of robotic-assisted surgery [39,40]. Further high-quality, risk-adjusted studies are warranted to clarify these outcomes. Similar to the early criticism surrounding LA, which eventually became normal surgery because of benefits like fewer infections, less discomfort, and shorter hospital stays, the current situation is similar [41-44].

Natural Orifice Transluminal Endoscopic Surgery

NOTES is a novel method that enables minimally invasive surgery via natural orifices such as the mouth, anus, or vagina. This treatment seeks to prevent noticeable scars on the body's surface. The concept of transvaginal appendectomy during vaginal hysterectomy was initially introduced in 1949 [45], with gynecologists performing it primarily for incidental appendectomy rather than treating AA [45,46]. Transvaginal appendectomy has three main approaches emerging: pure endoscopic (no abdominal ports), SILS-port assisted, and hybrid techniques utilizing umbilical ports. The treatment of gynecological diseases, not appendicitis, was the main goal of those studies, which did not include instances of AA. The first transvaginal appendectomy without vaginal hysterectomy was described by Palanivelu and colleagues from India in 2008 [47]. Three other cases were reported shortly after, one from Germany and two from Georgia. After dividing the mesoappendix and securing the appendiceal base with endoloops, surgeons carried out via endoscopic channels with snares or scissors. Notably, these cases employed a mere transvaginal endoscopic technique, relying on endoscopes without abdominal trocars or additional transvaginal instruments [48,49]. 

Operative times averaged 95 minutes (range, 72-135 minutes). Palanivelu and colleagues noted three prior conversions to LA due to technical challenges, while Bernhardt et al. emphasized their case involved subacute, not acute, appendicitis. NOTES offers possible advantages like decreased postoperative pain, shortened recovery periods, and decreased chances of incisional hernias, abdominal adhesions, and SSI by preventing visible scars. However, NOTES has a number of drawbacks and restrictions when compared to conventional approaches, including limited access and unusual operating angles and operative perspectives [47,48].

Emerging innovations

Needle-type Grasping Forceps for SILA

Between 2019-2020, SILA was introduced in adults employing a 1-cm umbilical incision, followed by two 5-mm trocars and needle-type grasping forceps inserted through McBurney's point. The appendix is dissected with an ultrasonic scalpel, then ligated and evacuated without contaminating the incision. Preliminary findings reported from 14 adult cases revealed no conversions, minimal discomfort (VAS 0-2), and short hospital stays (1-2 days) [50].

Endoscopic Retrograde Appendicitis Therapy

ERAT is an organ-preserving method of treating appendicitis that was introduced by Liu et al. in 2009 [51]. Unlike traditional appendectomy, ERAT uses a colonoscopy to access the appendix, remove obstructive fecaliths, and irrigate the lumen [52]. This minimally invasive procedure minimizes surgical scars, alleviates postoperative pain, and speeds up recovery. It is especially useful for high-risk individuals, such as pregnant women and young children [52,53].

Comparative outcomes of minimally invasive surgical techniques 

According to available data, LA has a number of advantages over open appendectomy (OA) for uncomplicated as well as complicated appendicitis. LA exhibits better results, such as fewer wound infections (presumably as a result of less wound contamination), in a meta-analysis of randomized controlled trials (RCTs), primarily including uncomplicated appendicitis in adults and children [54]. Additionally, despite higher operational times (11.59 minutes), which might correlate with surgical skill, it was linked to faster recovery, shorter hospital admissions (0.79 days), and fewer overall complications (Z=2.35; p=0.02) [54]. Although there were some concerns that LA may lead to more intra-abdominal abscesses, especially in patients who were perforated, meta-analyses reveal no discernible variation in overall abscess rates [55].

LA exhibits lower SSI (6.7% vs. 17.7%), earlier dietary resumption, and shorter hospital stays than OA, while maintaining comparable intra-abdominal abscess rates for mostly complicated appendicitis [56]. Although LA's superiority in pediatric instances has not been demonstrated, both patient groups benefit from its diagnostic advantages in equivocal cases [50,51]. Despite concerns regarding the best pediatric application, LA's clinical advantages, such as lower long-term risks of small bowel obstruction and special benefits for high-risk groups like obese patients, make it the recommended approach when expertise is available, even though cost-benefit analyses are required [57,58].

SILA, compared with CLA, is more technically challenging because of instrument crowding and less triangulation, even though both procedures exhibit comparable operational periods, blood loss, complication rates (such as wound infection, intra-abdominal abscess, and ileus), and postoperative pain levels [59-61]. Shorter hospital stays and better cosmetic results are two benefits of the surgery, but it also has higher conversion rates (18 SILA-to-CLA conversions compared to 3 CLA-to-open conversions) and may be more expensive because of the need for specialized equipment [59,62,63].

Notably, even in the lack of special wound protection measures, the meta-analysis did not support early worries regarding elevated wound infection risks with SILA [63]. It is important to take into account the challenging learning curve for SILA and the unanswered questions regarding the potential risks of umbilical hernias. Despite its greater technical requirements and expenses may prevent broad use until further standardization takes place, these findings present SILA as a viable alternative to CLA for a select number of patients when carried out by skilled surgical teams [59,62,64].

Although pure endoscopic NOTES techniques showed promise, their lengthy operating times (72-135 minutes) and technical complexity prevented widespread use [65]. According to a meta-analysis, hybrid NOTES, compared to traditional laparoscopy, exhibited considerably reduced postoperative pain levels in the first week. This is probably because it avoids making incisions on the abdominal wall [66]. RCTs did not support the benefits that non-randomized research reported, such as reduced complication rates and shorter hospital stays, which may indicate selection bias in observational studies [67-69]. Crucially, when surgeon experience was taken into consideration, RCTs revealed similar safety profiles across the procedures, with no discernible variations in major morbidity, surgical time, or hospital stay [70,71]. Though cultural factors and a lack of data in cases of morbid obesity and difficult appendicitis remain major barriers to wider deployment, current evidence supports its use in non-obese (BMI<35) patients with uncomplicated appendicitis [72].

Robotic surgery, compared to LA, showed better postoperative recovery outcomes. These outcomes included shorter hospital stays and a quicker return to normal activities. It should be noted that LA is more accessible than the robotic approach, and hence it continues to hold its place as a dependable, affordable substitute with demonstrated advantages over open surgery, such as less postoperative pain and earlier recovery. However, compared to laparoscopic or open surgery, robotic surgery is still very expensive, which is one of its main disadvantages. RAA requires specialized training, and the expense of purchasing, utilizing, and maintaining a surgical robotic system is significantly more than that of traditional surgical techniques. The restricted availability, need for specialized training, and expensive equipment prices provide further difficulties for the robotic approach. Although there are limited available RCTs on RA, the information now available suggests that LA is still the more sensible option in the majority of situations [73].

In 2019, SILA with needle-type grasping forceps (SLAN) was introduced in pediatric cases and showed beneficial results such as less trauma, faster recovery, and less scarring [74]. Evidence currently available shows that ERAT and LA have different benefits and drawbacks. While retaining similar safety profiles to laparoscopic surgery, ERAT shows better results in terms of operational duration (shorter operation time) and cost-effectiveness (about $600 less each procedure) [75,76]. LA, on the other hand, has far lower recurrence rates (14.25 times lower chances of appendiceal disease recurrence) and much higher technical success rates (5.46 times larger odds of completion). Notably, stented cases do not exhibit any more episodes throughout follow-up periods, suggesting that stent implantation during ERAT is essential for preventing recurrence. According to these results, laparoscopy is still the more dependable method for conclusive therapy; however, ERAT might be better in certain situations where appendix preservation is important [76].

Patient selection and perioperative considerations 

AA is the most frequent surgical emergency presenting as an acute abdomen. Accurate and timely diagnosis is essential, as delayed intervention can lead to complications such as perforation, abscess formation, or sepsis [77,78]. Proper patient selection is therefore critical for the safe and effective use of minimally invasive surgical techniques.​​​​​​

Risk stratification tools, such as the Alvarado scoring system [79,80], and imaging modalities, including ultrasound and computed tomography (CT) [81-84], can support surgical decision-making by identifying patients who may benefit most from immediate intervention versus those suitable for conservative management or delayed surgery. These diagnostic considerations help optimize the timing and approach of minimally invasive procedures, potentially reducing unnecessary interventions and associated postoperative complications [78].

Special populations

Pediatric Patients

AA is still difficult to diagnose in children, especially in preschoolers who frequently show greater rates of complications and rapid progression. In the pediatric age group, the Alvarado score and the Pediatric Appendicitis Score (PAS) both have insufficient discriminatory power and overestimate AA diagnoses [85]. The Appendicitis Inflammatory Response (AIR) score, which includes C-reactive protein (CRP) and pain rating, performs better than the aforementioned scores [86]. Novel approaches such as Bonadio's perforation risk score and the Pediatric Appendicitis Laboratory Score show promise in risk stratification and ruling out AA [87,88].

Also in pediatrics, ultrasound is considered the first-line imaging modality. However, its accuracy varies depending on the operator and decreases as BMI increases [89]. Staged algorithms that use low-dose CT (sensitivity 95.5%, specificity 94.9%) or magnetic resonance imaging (MRI) (sensitivity 96.5-97.4%, specificity 94.3-97.1%) for inconclusive cases maximize diagnostic yield while reducing radiation exposure [90]. MRI is especially useful for differentiating perforated AA and lowering negative appendectomy rates [82]. However, its cost might limit its widespread use. When paired with clinical scores, biomarkers such as procalcitonin, calprotectin, and CRP improve diagnostic accuracy and allow for safer conservative care in low-risk situations [82].

Non-operative management (NOM) with antibiotics shows equivalent safety to surgery for the majority of children with uncomplicated AA (≥81%), with success rates ranging from 58 to 100% and lower morbidity, fewer disability days, and lower costs [91-93]. The presence of an appendix considerably raises NOM failure rates (47-60%), and recurrence rates vary (0.1-31.8% at 1 year) [82,94,95]. Meta-analyses support the viability of NOM but point out trade-offs: readmission risks are higher (RR 6.98), especially in the absence of appendicolith exclusion, even though complications are comparable (0-13% vs. 0-17% for surgery) [96,97]. According to Minneci et al.'s cohort study, which demonstrated NOM's benefits in fewer disability days (2.7% vs. 12.3% complex AA) and expenses, patient-specific factors affect results [91].

According to available data, NOM is a good choice for a limited percentage of pediatric patients without appendicoliths, as long as families are informed of the possibility of failure (14% recurrence) and the slight chance of undetected complicated AA [91,92]. Systematic reviews place an emphasis on shared decision-making [96]. Guidelines reserve surgery for high-risk cases, such as those with appendicoliths or diagnostic ambiguity, and conditionally advocate NOM [82,97]. Although more RCTs are required to improve patient selection criteria, this customized strategy provides an appropriate balance between efficacy and safety.

In children, CLA is safe and effective in reducing SSIs and bowel obstructions compared to OA but may carry a slightly increased risk of intra-abdominal abscess (IAA), highlighting the need for a careful approach and selection based on patient-specific factors [98]. According to a meta-analysis by Zhang and colleagues, the incidence of overall postoperative problems, IAA, ileus, wound hematoma, length of hospital stay, and frequency of use of extra analgesics did not significantly differ between SILA and CLA. However, SILA needed a longer operating time and was linked to a higher incidence of SSI than three-port LA [99].

Pregnant Women

When considered as standalone criteria, almost all clinical signs and symptoms cannot substantially differentiate pregnant women with and without AA. While current scoring systems like Alvarado show decreased accuracy in pregnancy due to physiological changes (sensitivity 78.9%, specificity 80%), the Adult Appendicitis Score (AAS) performs the best among risk prediction models for women (specificity 63.1%, failure rate 3.7%). Among available imaging options, MRI is recommended as the most reliable imaging modality, with meta-analyses demonstrating 90.5-94% sensitivity and 97-98.6% specificity. Nonetheless, non-visualization rates can reach 30-43% in later pregnancy [100-103]. Although ultrasonography is still the preferred first-line imaging modality, it has serious drawbacks, including non-visualization rates ranging from 34.1% to 71%, which are later detected by MRI [104,105].

After an inconclusive ultrasound, current guidelines support MRI. However, there is increasing evidence that MRI may be a better option for the first line of treatment due to its superior diagnostic performance and lack of radiation risks, especially for women of reproductive age, where it has 100% specificity and 96-100% negative predictive value [106-108].

In addition to providing therapeutic benefits, such as shorter hospital stays and fewer SSI, LA during pregnancy shows fetal safety that is comparable to that of OA [82]. Recent meta-analyses (n=4,694 patients) indicated no significant difference in fetal loss or preterm delivery rates after omitting an outlier study, despite earlier studies raising concerns about increased fetal loss with LA [109]. Evidence currently available indicates that LA should be chosen over OA in pregnancy, where surgical skill is available [82].

Complicated appendicitis

For managing complicated AA, LA outperforms the open approach in children, with significantly reduced SSI rates and shorter hospital stays. Additionally, the procedure enhances quality of life and lessens postoperative pain, making it recommended as long as expertise exists. As meta-analyses of more than 2500 patients reveal no decrease in IAA or SSIs, while adding roughly seven minutes to the surgical time, the current evidence disproves the usual use of peritoneal irrigation during LA. For the management of intra-abdominal collections, suction alone is now advised [82,110].

Furthermore, there are no therapeutic advantages to either the abdominal drainage or stump closure approach. Drains lengthen fasting durations, antibiotic use, and recuperation periods, according to pediatric studies. Large systematic reviews (n=5934) suggest that drainage increases hospital stays by 2.17 days without lowering IAA or SSI risks [111]. The only independent risk factor for the development of IAA is still complicated appendicitis, which highlights the necessity of standardized surgical techniques as opposed to supplementary therapies like irrigation or drainage [112,113].

Global perspectives and health systems 

Minimally invasive surgery is nowadays considered by high-income countries (HICs) as a standard treatment for a variety of surgical operations. Minimally invasive surgery adoption is still restricted in low- and middle-income nations (LMICs) as these countries have the most common widely performed technique as open surgery, especially in public hospitals and rural locations with limited access to contemporary equipment [13,14]. Financial constraints stand as significant obstacles due to the high cost of purchasing and maintaining laparoscopic equipment. Furthermore, a shortage of skilled workers and maintenance support worsens the situation. The discrepancy also exists in surgical training and education. Trainee exposure to minimally invasive surgery is low in several LMICs, especially in East, Central, and Southern Africa.

According to research by the College of Surgeons of East, Central, and Southern Africa (COSECSA), laparoscopic procedures accounted for just 0.9% of trainees' logged procedures [114]. This also further demonstrates how little minimally invasive surgery is used in LMICs and the gaps in their infrastructure and modern procedures. Some initiatives being utilized to bridge this gap include telementoring, low-cost simulation technologies, and partnerships with commercial institutions, which often have better infrastructure and international support. Two developments that could increase the use of minimally invasive surgery in LMICs are gasless laparoscopy and reusable instruments. These solutions require strong infrastructural investment and coordinated global health efforts to ensure sustainability and equal access [114].

Future directions

The landscape of minimally invasive surgical techniques for appendicitis is continuously evolving, driven by advancements in technology and a deeper understanding of patient outcomes. While current laparoscopic approaches have significantly improved patient care compared to open appendectomy, several avenues for further innovation and refinement are emerging. These future directions aim to enhance surgical precision, reduce invasiveness, improve cosmetic results, and broaden accessibility, particularly in resource-limited settings.

One significant area of development is endoscopic appendectomy, which represents a paradigm shift towards truly scarless surgery. This non-invasive technique involves accessing the appendix through natural orifices, such as the mouth or vagina, eliminating external incisions. Early studies demonstrate its feasibility and potential advantages, including superior cosmesis and reduced incisional complications like hernias and infections [115]. However, widespread adoption is currently limited by the technical complexity, the need for specialized training, and the absence of standardized guidelines for patient selection and procedural techniques. Future research will focus on refining these endoscopic approaches, developing more intuitive instrumentation, and establishing clear protocols to ensure safety and efficacy across diverse patient populations.

Artificial intelligence (AI) is poised to revolutionize surgical practice, including appendectomy, by enhancing diagnostic accuracy, guiding intraoperative decision-making, and optimizing postoperative management. AI algorithms, particularly those leveraging machine learning, have shown promise in accurately diagnosing AA, often outperforming traditional scoring systems [116]. In the operating room, AI-guided systems could provide real-time anatomical mapping, identify critical structures, and even predict potential complications, thereby increasing surgical precision and reducing errors. Further integration of AI into surgical platforms will require robust validation through large-scale clinical trials and the development of ethical frameworks to ensure responsible implementation [117].

Another promising area involves the development of next-generation innovations such as biodegradable materials. The use of biodegradable ports and other surgical instruments could, in theory, eliminate the need for secondary procedures for removal and potentially reduce long-term complications associated with retained foreign bodies. While research in this specific application for appendectomy is still speculative, advances in biodegradable materials across various surgical specialties suggest potential benefits, such as reduced pain, improved wound healing, and a lower risk of port-site hernias. Further studies are required to establish biocompatibility, mechanical integrity, and degradation profiles suitable for appendectomy [118].

Finally, addressing research gaps remains crucial for advancing minimally invasive appendectomy. Long-term outcomes of newer techniques like SILA and NOTES need further investigation to fully understand their benefits and potential drawbacks over extended periods. Cost-effectiveness studies, particularly in LMICs, are essential to ensure that these advanced techniques are not only clinically superior but also economically viable and accessible globally. Validation of emerging therapies, such as ERAT, through rigorous randomized controlled trials is also critical to establish their role in the management algorithm of appendicitis.

The future of minimally invasive appendectomy is characterized by a drive towards less invasive techniques, intelligent surgical assistance, and innovative biomaterials. These advancements, coupled with continued research into long-term outcomes and global accessibility, hold the potential to further improve patient care and redefine the standard of appendicitis management worldwide.

## Conclusions

Minimally invasive techniques, particularly LA, continue to offer clear advantages over open surgery in reducing postoperative pain, hospital stay, and surgical site infections. However, outcomes remain dependent on patient selection, diagnostic accuracy, surgeon expertise, and resource availability. Emerging approaches such as SILA, RAA, and ERAT show promise but require further evidence due to technical, financial, and infrastructural limitations. Diagnostic scoring systems and staged imaging pathways remain important for reducing unnecessary surgery, though none are universally definitive. Future progress in appendicitis management will rely on generating stronger comparative evidence, improving training, and addressing global disparities in access to minimally invasive care.
